# Neural network-based analysis algorithm on Mueller matrix data of spectroscopic ellipsometry for the structure evaluation of nanogratings with various optical constants

**DOI:** 10.1515/nanoph-2024-0565

**Published:** 2025-02-12

**Authors:** Juwon Jung, Nagyeong Kim, Kibaek Kim, Jongkyoon Park, Yong Jai Cho, Won Chegal, Young-Joo Kim

**Affiliations:** Department of Mechanical Engineering, 26721 Yonsei University, Seoul, Republic of Korea; Semiconductor and Display Metrology Group, Strategic Technology Research Institute, Korea Research Institute of Standards and Science, Daejeon, Republic of Korea; Semiconductor and Display Metrology Group, Korea Research Institute of Standards and Science, 267 Gajeongno, Yuseong-Gu, Daejeon, 34113, Republic of Korea; Graduate School of Analytical Science and Technology (GRAST), Chungnam National University, Daejeon, 34134, Republic of Korea

**Keywords:** spectroscopic ellipsometry, neural network, optical constant, nanostructure

## Abstract

Accurate and fast characterization of nanostructures using spectroscopic ellipsometry (SE) is required in both industrial and research fields. However, conventional methods used in SE data analysis often face challenges in balancing accuracy and speed, especially for the *in situ* monitoring on complex nanostructures. Additionally, optical constants are so crucial for accurately predicting structural parameters since SE data were strongly related to them. This study proposes a three-step algorithm designed for fast and accurate extraction of structural parameters from SE measurements. The method utilizes three neural networks, each trained on simulation data, to obtain optical constants and progressively refine the prediction on structural parameters at each step. When tested on both simulation and measurement data on the fabricated 1D SiO_2_ nanograting specimen, the algorithm demonstrated both high accuracy and fast analysis speed, with average mean absolute error (MAE) of 0.103 nm and analysis speed of 132 ms. Also, the proposed algorithm shows more flexibility in accounting for any change of optical constants to serve as a more efficient solution in the real-time monitoring.

## 1 Introduction

Spectroscopic ellipsometry (SE) is a powerful technique for analyzing nanostructures to comprehensively understand the optical constants of materials and structural characteristics at the nanoscale. It measures how the polarization of light changes upon reflection from a specimen. By analyzing this change, SE can estimate the optical properties and structural parameters of the specimen [1], [2], [3], [4], [5], [6]. As a nondestructive method, SE has contributed to advancing research in semiconductor manufacturing for thin films [7], [8], two-dimensional (2D) nanomaterials [9], [10], biophotonics [11], and nanofabrication [12]. In spite of its many advantages, technical difficulties related to the application of SE to complex nanostructures persist, particularly in terms of the data analysis speed. In analytical equipment, iterative-process-based nonlinear regression data-fitting methods are used to obtain the fitting parameter values that correspond most closely to the observed data because the determination of optical constants and structural parameters is an inverse problem. Moreover, for the analysis of nanostructures other than thin films, these methods require numerical optical simulations, such as the rigorous coupled wave analysis (RCWA) method [13], finite-difference time-domain (FDTD) method [14], and finite element method (FEM) [15]. These simulation-based methods are executed iteratively, which increases the overall analysis time substantially. This limitation is more apparent in scenarios where rapid analysis and high throughput are needed in nanostructure characterization [16].

For high-speed SE data analysis, various approaches have been proposed to reduce analysis time by minimizing the reliance on iterative fitting and simulation calculations [17], [18]. One such approach involves using pregenerated library data in conjunction with a search algorithm, such as including K-dimensional tree [19], heuristic search [20], and correction-based library search [21], to find data that are the most similar to the measured data. The objective is to simplify the search process to ultimately reduce the analysis time for the measured data. Furthermore, recent studies have explored the integration of artificial intelligence (AI) with ellipsometry to expedite data analysis and increase prediction accuracy [22], [23], [24], [25]. For example, Yann et al. demonstrated that the accuracy of ellipsometric measurements based on artificial neural network (ANN) analysis is comparable to that of energy-dispersive X-ray spectroscopy, a classical alloy stoichiometry characterization tool [26]. Jiang et al. reported that by utilizing deep neural networks in the analysis process, they achieved a level of accuracy comparable to RCWA-based analysis and significantly accelerated the analysis speed [27]. In addition, compared to conventional spectroscopic ellipsometry (SE), MMSE allows for the measurement of additional Mueller matrix components for samples with anisotropic optical properties. Thus, it is expected that the severe correlation between structural parameters can be reduced when MMSE data were used in the analysis model for the nanostructure patterned samples [28].

Building upon these advancements, we have also already proposed a method that uses Mueller matrix spectroscopic ellipsometry (MMSE) to analyze nanograting structures [6]. This approach comprises two main steps: The first step involves matching the measured Mueller matrix (MM) data to the closest node in the preacquired MM library dataset, and the second step entails using an ANN to predict the structural parameters by combining the precalculated parameters locally near the closest node. In that study, we used the Levenberg–Marquardt (LM) method in the first step to iteratively match the closest node for improving accuracy. However, the LM method requires a longer analysis time for applying the large volume of library data, especially as the number of structural parameters increases. Moreover, it is also required to consider variable optical constants in the analysis algorithm since they are affected by the processing conditions and temperature variations [29], [30], [31].

In this study, thus we aim to develop an ANN-based analysis algorithm capable of accurately analyzing structural parameters of nanostructured specimens without repreparing the training dataset for actual optical constants of the material. In addition, the previous least-square iterative computation was replaced with neural network computation for high-speed analysis. The final analysis algorithm comprises three distinct steps, each utilizing a dedicated neural network. In the first step, the measured MM is input into a neural network for the prediction of optical constants that predicts wavelength-dependent data. This neural network converts the measured MM into the matched one corresponding to the specimen with identical structure but prefixed optical constants, while simultaneously predicting the optical constant of the measured specimen. In the second step, the converted MM is used to select the node point in the parameter space that is the closest to the parameter position of the actual specimen assuming fixed intervals in the parameter space of the specimen. In the third step, the library data corresponding to the two selected points and converted MM are analyzed simultaneously to predict the structural parameters of the specimen. For the experimental evaluation of our proposed analysis algorithm, we fabricated an SiO_2_ one-dimensional (1D) grating structure with a width of 38 nm and analyzed its optical constants and structural parameters.

## 2 Methodology

### 2.1 Three-step-analysis algorithm for optical constants and structural parameters

**Figure 1:**
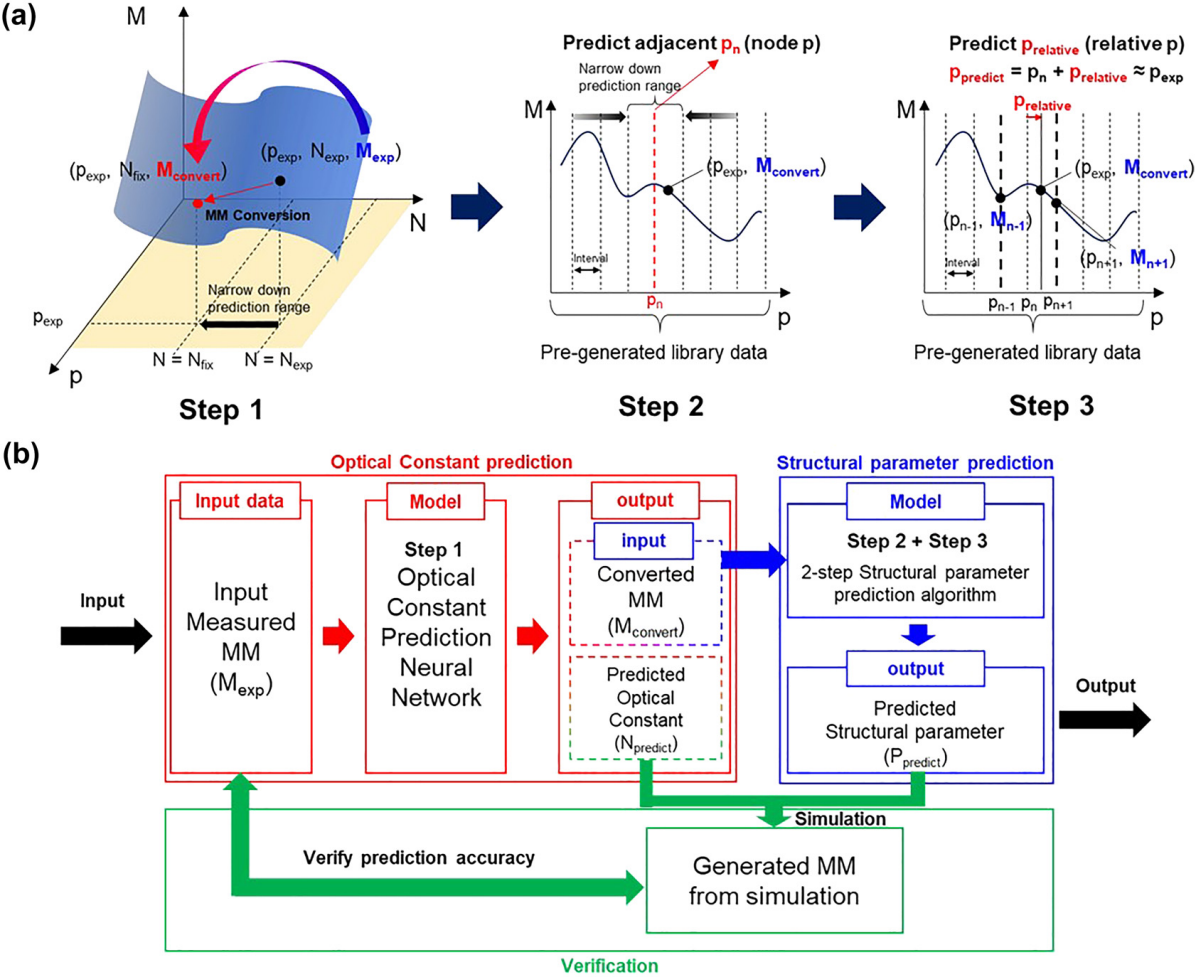
Overview of the three-step analysis. (a) Predicted data according to the input data for each step of the analysis. (b) Schematic of the proposed three-step analysis algorithm.

Spectroscopic ellipsometer (SE)-based analysis of specimens is primarily performed to extract both the structural parameters and optical constants of the nanostructure. The Mueller matrix (MM) is a 4 × 4 real matrix, and it contains data that represent the interaction between the specimen and the applied electromagnetic field, and it is influenced by the three factors: incident light conditions (incident angle and azimuthal angle), structural parameters, and optical constants of the nanostructure. Assuming the incident light condition is constant, the other two factors are considered the main variables. The optical constants can be expressed as vectors of multiple elements. Similarly, the structural parameters of the nanostructure and the MM can be expressed as vectors of multiple elements. When simplified and represented graphically, as depicted in Figure 1(a), the MM can be plotted on the *x*- and *y*-axis coordinate system as a function of structural parameters (*p*) and optical constants (*N*), including the refractive index (*n*) and extinction coefficient (*k*). The primary goal of our analysis algorithm is to determine both optical constants (*N*_exp_) and structural parameters (*p*_exp_) of the unknown specimen from the measured MM (*M*_exp_). For the step-by-step analysis, we divided the algorithm into 3 sequential neural network-based steps.

In the Step 1, the measured MM (*M*_exp_) is matched to the converted MM (*M*_convert_) having the same structural parameters (*p*_exp_) but the prefixed optical constants (*N*_fix_) using newly developed algorithm. Simultaneously, the optical constants of the nanostructure material can be predicted based on the lots of training data having a range of optical constants and structural parameters. Then, the *N*_fix_ are selected to accurately predict the structural parameters (*p*_exp_) without considering the variation of optical constants in the following steps. By converting the *M*_exp_ into the *M*_convert_, the prediction parameter is reduced from the both *p* and *N* axes to the *p* axis only, as shown in Figure 1(a). In the Step 2, after dividing the *p*-axis into specific intervals and defining several node points, the *M*_convert_ is analyzed to select the matching node (*p*_*n*_) that is the closest to the *p*_exp_. The prediction range for the *p*_exp_ can be then narrowed between the two adjacent nodes (*p*_*n*−1_, *p*_n+1_). Finally, in the Step 3, the position (*p*_exp_) is predicted exactly in the narrow range by analyzing the *M*_convert_ along with the preacquired MM data on the two adjacent nodes through simulation. If the MM data of each node point are pregenerated as library data through simulation, the node at which all the parameters are one gap ahead of the *p*_*n*_ output in the previous step is defined as +1 order node (*p*_*n*+1_). Conversely, the node at which all the parameters are one gap behind the *p*_*n*_ output in the previous step is defined as −1 order node (*p*_*n*−1_). Thus, the library MM data corresponding to these two defined nodes (+1- and −1-order nodes) are input together with the *M*_convert_ to output *p*_predict_.

The architecture of our proposed three-step analysis algorithm is presented in Figure 1(b). Firstly, the measured MM data are used as input to simultaneously predict the optical constants of the specimen materials and the *M*_convert_, which has the same structural characteristics and *N*_fix_ properties. Since the optical constant characteristics of the specimen were obtained, we call this step as optical constant prediction algorithm. In next steps, the structural parameters for the *N*_fix_ are extracted, and these two steps constitute what we call the structural parameter prediction algorithm.

This three-step analysis algorithm is not an iterative process, and the structural parameters are predicted using three neural network calculations, allowing for fast analysis at the millisecond level for each data point. Additionally, the structural parameter can be predicted accurately even when the optical constants are changed owing to the different material selection or any effects of environmental conditions. Furthermore, the newly developed algorithm can allow for the reverification of the predicted parameters by using the final optical constants (*N*_predict_) and structural parameters (*p*_predict_) as new inputs for the RCWA simulation. The resulting simulated MM data can be compared again to the measured MM to verify the reliability of the predicted structural parameters, as explained in the green box in Figure 1(b). In the following sections, we will explain the 1D grating model for the nanostructure evaluation with both prediction algorithms in detail on the optical constants and structural parameters, respectively.

### 2.2 Model for 1D-grating periodic structure and measurement conditions

**Figure 2:**
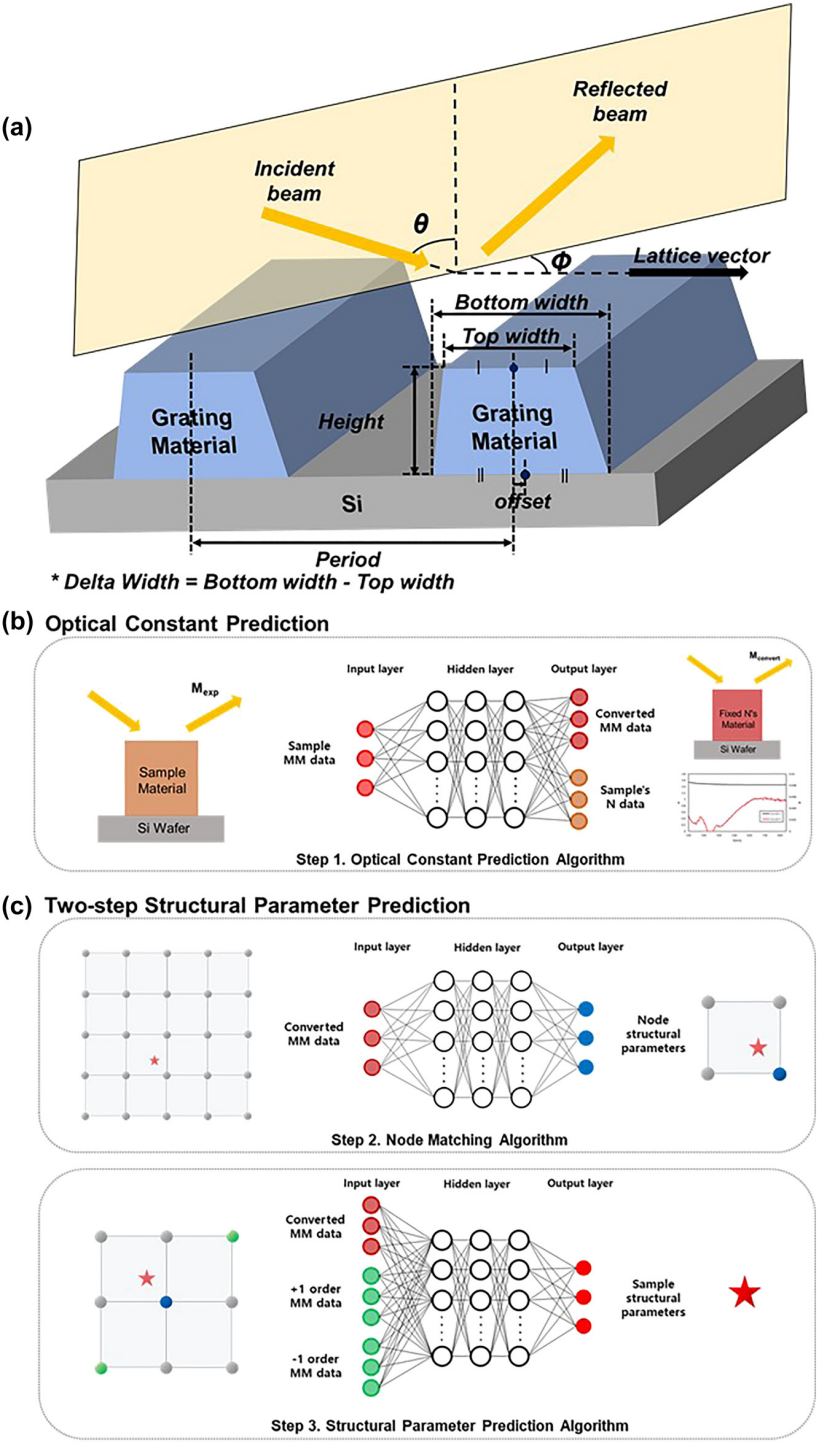
Definition of structural parameters: (a) Schematic structure of 1D grating specimen with the definition of structural parameters. Framework of the proposed three-step analysis algorithm: (b) optical constant prediction algorithm and (c) structural parameter prediction algorithm.

We use a 1D grating nanostructure specimen with nanoscale variations, as illustrated in Figure 2(a), to evaluate the proposed algorithm for predicting four structural parameters, namely height, average width, delta width, and offset. Height refers to grating height, average width is the average of the top width and bottom widths, delta width is the value obtained by subtracting the top width from the bottom width, and offset is the relative position obtained when using the lattice vector of the 1D-grating as the axis, as calculated by subtracting the center point of the top width from the center point of the bottom width. If the center point of the bottom width is positioned in the positive direction of the axis compared to the center point of the top width, the offset is positive value; else, it is negative value. Then, we set the prediction range for the structural parameters and acquire training data in this range. The height of the structure is 80–120 nm, average width is 30–45 nm, delta width is 0–15 nm, and offset is −10–10 nm. Notably, nonreasonable physical ranges of the grating structure are excluded from this analysis. It was also assumed that the 1D grating material has a range of optical constants, such as a refractive index from 1.25 to 1.70 and the absorption coefficient from 0 to 0.2 at a 555 nm wavelength, based on the measured data from available various materials.

Finally, the MM data measured through SE have the following light conditions, where the angle of incidence is fixed at 70°. The azimuthal angle, which is the angle between the plane of incidence and the lattice vector of the 1D-grating, is set to two values: 0° and 45°. The data obtained for one incidence angle and two azimuthal angles are used together as the measurement data for analysis. The wavelength range is set to 220–858 nm in steps of 2 nm. All training, validation, and library data were generated by performing an RCWA simulation.

### 2.3 Optical constant prediction algorithm

As described in Section 2.1, the optical constant prediction algorithm converts the measured MM corresponding to the *N*_fix_ to focus the prediction dimension solely to structural characteristics in the following steps. Given the measured MM as the input, the algorithm outputs a *M*_convert_ corresponding to a nanostructure with the same structural parameters (*p*_exp_) but *N*_fix_, and simultaneously predicting the optical constants (*N*_predict_) of the specimen material. This neural network-based algorithm predicts optical constants as a function of wavelength as shown in Figure 2(b). The optical constants of grating materials used in the training were selected from a database of the optical constants of publicly available materials, which covers the wavelength range of 220–858 nm. Materials with *n*-values between 1.25 and 1.7 and *k*-values between 0 and 0.2 at a 555 nm wavelength were considered in this study, as summarized in Table 1. Each data case was generated using combinations of the optical constants of the grating material and its structural parameters. The structural parameter cases were drawn from the set of cases (*k* cases) used in the node-matching algorithm, while the optical-constant cases were selected from the publicly available database that fulfills the defined range criteria (l cases). Because the total number of combinations (*k* × l cases) would be too large and analyzing all of them would be computationally inefficient, a sampling method was used to select a subset of cases.

**Table 1:** Distribution of *n* by range when *λ* = 555 nm of optical constant cases.

*n* value at *λ* = 555 nm	The number of cases for train	The number of cases for test
1.25–1.30	3	
1.30–1.35	9	2
1.35–1.40	11	
1.40–1.45	11	1
1.45–1.50	18	3
1.50–1.55	6	
1.55–1.60	3	1
1.60–1.65	1	
1.65–1.70	1	

Given that we aimed to accurately predict *p*_exp_, we generated datasets for all structural parameters with various optical constant cases. For each loop, targeting all structural parameter cases, we sequentially sampled one optical constant case from the set of optical constant cases for each structural parameter and generated individual training data points for each case. This process was repeated for multiple sampling loops, and in this manner, we obtained several datasets by increasing the number of sampling loop (*m*) starting from *m* = 1. Subsequently, each of these datasets was trained on the same neural network architecture, and the performance levels of the trained models were evaluated individually. When the simulated MM for a specific *N*_exp_ and *p*_exp_ was input, learning was performed by minimizing the difference between the output value and the label value, where the MM corresponding to a specific *N*_fix_ and *p*_exp_, along with *N*_exp_, were used as label values.

The trained neural network, when provided with the measured MM, output the *M*_convert_ within the specified wavelength range and the *N*_predict_. The *M*_convert_ will then be an input into the structural parameter prediction algorithm, while the *N*_predict_ is used as an input for simulation and verification alongside the *p*_predict_ (see Figure 1(b)).

### 2.4 Structural parameter prediction algorithm

In our previous research [6], we proposed an algorithm to predict structural parameters through a two-step process. When the library data of each node in a specific interval within a specific range are pregenerated, the node-matching algorithm, which is the first of the two steps, uses Levenberg–Marquardt (LM) optimization to select the *p*_*n*_ with the smallest difference relative to the MM in the library by comparing it to the measured MM provided as the input. In the next step, the structural parameters were predicted after finding the relative position of *p*_exp_ with respect to *p*_*n*_ by inputting the MMs corresponding to the +1- and −1-order nodes, along with the measured MM, into a trained neural network. Here, a relatively wide interval of 5 nm was set to create a limited amount of library data, and find the closest library MM data with the measured MM for narrowing the prediction range in the next step. However, as the number of predicted parameters increases, the time required for searching increases exponentially, leading to an increase in the computation time.

In this paper, we retain the structural parameter prediction algorithm but introduce new node matching neural network algorithm to instantly predict *p*_*n*_ by training lots of presimulated MM data, rather than iteratively comparing the measured MM to the library MM to find the closest MM. To predict four parameters, the structural parameter can be represented as a vector *p* = {*p*_height_, *p*_averagewidth_, *p*_deltawidth_, *p*_offset_}, and the library data are generated in the parameter space in which the four predicted parameters form axes at intervals of 5 nm within a predefined range. The nodes at the intersections of these intervals are pregenerated through simulations and stored as library data. As in our previous study [6], “data density,” which refers to the number of training data points within each “unit cell” formed by the smallest interval of each parameter axis, is set to 7. The training data and library data are generated by performing an RCWA simulation of a 1D grating structure, in which the vector *p* and an *N*_fix_ for SiO_2_ are considered.

In this newly developed node-matching algorithm, for each training data point MM based on the data density, the vector *p* of the library node with the shortest Euclidean distance to *p*_exp_ in four-dimensional space is labeled the training target (see Figure 2(c) upper panel). In the structural parameter prediction algorithm, after matching each training data point obtained according to the data density to each node located at all vertices of the unit cell, the MMs of the two library nodes corresponding to the +1 order and −1 order of the matched node and the measured MM are used as the training data. These data are combined and input into the neural network. Then, training is performed by setting the relative position of *p* in the training data compared to that of *p* in the matched node as the label data. Because this approach generates training data by combining all the vertex nodes for each training data point, a larger amount of training data can be obtained than that possible with the node matching algorithm (see Figure 2(c) lower panel).

After training both neural networks separately, when *p* is close to the boundary of the prediction range during the analysis, there exists a possibility of matching the closest node to the boundary. If this situation occurs and the selected node is a boundary node where either the +1 node or −1 node MM does not exist, all the boundary parameters are shifted inward by one interval, allowing access to the +1 or −1 node library data. This step ensures that all the *p*_exp_ within the prediction range can be analyzed.

## 3 Evaluation results

### 3.1 Training of neural network model

The library data consist of the MM data generated for all nodes within the defined parameter range, with intervals of 5 nm between nodes, totaling to 1,302 data points. The training dataset for Step 1 was generated by combining the structural parameter cases with the optical constant cases from the optical constant database that met specific conditions. For the structural parameter cases, simulation data were generated, including 5,278 training data, 660 validation data, and 660 test data in 8:1:1 ratio through simulations, after applying a data density of 7 within the prediction range. For the optical constant cases, total of 70 cases were identified from the database and split into 63 training cases and 7 test cases in 9:1 ratio. Table 1 shows the distribution of the 63 training and 7 test optical constant cases. The training data were generated by sampling randomly from the 63 optical constant training cases (*N*) for each of the 5,278 structural parameter training case (*p*). MM data were then produced through simulations using the sampled *N* and *p* as inputs. The dataset was generated by incrementing the number of sampling loop (*m*) from *m* = 1, with neural network training conducted on each resulting dataset. For example, for *m* = 1, the number of training data points was 5,278 (5,278 × 1), and for *m* = 2, the number of training data points was 10,556 (5,278 × 2). Datasets were generated by increasing m from 1 to 30, and each was used to train the neural network. The validation and test datasets were constructed by sampling from the 7 test optical constant cases for each structural parameter’s validation and test cases, generating the corresponding MM data through simulations using the selected *p* and *N* as inputs. For this process, both the validation and test sampling loops were set to 1, ensuring consistent use of these datasets across all trained neural networks.

The neural network model used in Step 1 was Swin U-Net, which is known for its powerful performance in medical image segmentation [32]. This neural network captures the global context effectively, models complex spatial relationships, and processes images of varying sizes. Swin U-Net includes both an encoder and a decoder, resulting in a symmetric architecture. In terms of hyperparameters, the arbitrary dimension, window size, number of layers, and attention heads were set to *C* = 48, *W* = 20, *L* = 14, and *h* = 6, respectively, as the defaults. The input dimension for Step 1 was 320 × 15 × 2 because the MMs measured for two azimuthal angles were used, and the output dimension was 320 × 16 × 2 for predicting the *M*_convert_ and *N*_exp_ (*n*, *k*). The neural network was trained by providing the simulated MM for *p*_exp_ and *N*_exp_ as the input and performing training to predict the MM for *p*_exp_ and *N*_fix_ (by using the MM from the dataset of Step 2 for *N*_fix_) as well as *N*_exp_ (*n*, *k*) as the label.

For the dataset of the neural network in Step 2, Step 1’s structural parameter cases were used, including 5,278 training data, 660 validation data, and 660 test data. The dataset for training the neural network in Step 3 was generated by matching each individual data point to all the vertices of the unit cells in the parameter space. This operation yielded a dataset consisting of 30,598 training data, 3,938 validation data, and 3,527 test data.

The neural networks used in Step 2 and Step 3 were based on the Swin Transformer architecture, which performs well in handling complex computer vision tasks [33], [34]. Similar to Swin U-Net, Swin Transformer captures both global and local contexts through its hierarchical structure; however, it includes only the encoder component, unlike Swin U-Net’s encoder–decoder setup. This model efficiently captures global and local contexts through its hierarchical structure, making it effective at extracting structural parameter features in the local and global regions of the MM data across wavelengths. The hyperparameters of the Swin Transformer for both Step 2 and Step 3 were identical to those used in Step 1. The key difference between Step 2 and Step 3 was in the input dimensions: In Step 2, only the *M*_convert_ was used as the input, resulting in an input dimension of 320 × 15 × 2 considering the two azimuthal angles. By contrast, in Step 3, the *M*_convert_ and the +1- and −1-order node library MMs were used as the inputs, resulting in an input dimension of 320 × 15 × 6. The output dimension was 4 × 1 for both steps because it predicts four structural parameters, and training was conducted to minimize the difference between the predicted output and the label. All ANN models were trained using an Intel Xeon W-2245 CPU at 3.90 GHz with 64 GB of memory, alongside an NVIDIA RTX A5000 GPU.

### 3.2 Evaluation for the prediction algorithm

**Figure 3:**
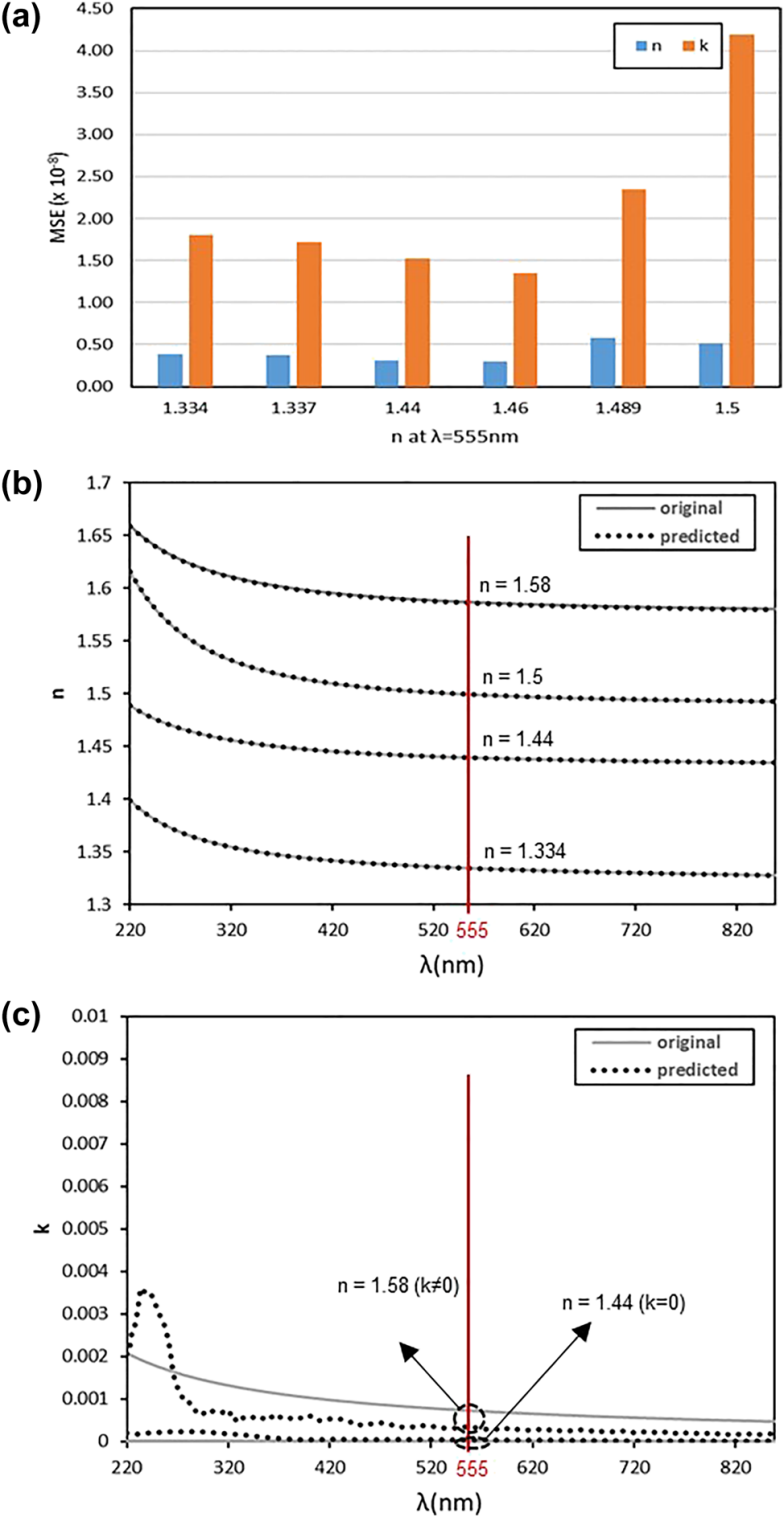
Evaluation results of optical constant prediction performance for 660 test data: (a) MSE of *n* and *k* of 6 test materials. (b) Predicted refractive index (*n*) based on the optical constants of 4 test materials, (c) predicted extinction coefficient (*k*) based on the optical constants of 2 test materials according to *n* at *λ* = 555 nm.

The proposed algorithm consists of three individually trained neural networks, each evaluated separately for its performance. The purpose of the Step 1 neural network was to output a *M*_convert_ close to the one corresponding to *N*_fix_ and *p*_exp_ while simultaneously predicting *N*_exp_. We evaluated the prediction accuracy for *N*_exp_ by using a model trained with a dataset where the sampling loop count (*m*) was set to 30. Specifically, for six optical constant cases with *n* values at a 555 nm wavelength, we compared the mean square error (MSE) of the predicted *n* and *k* values for each case, as shown in Figure 3(a). In Figure 3(a), both *n* and *k* exhibit the lowest MSE for cases with *n* = 1.46 with *k* = 0, while the MSE increases as *n* changes further away. As shown in Figure 3(b) and (c), the prediction error for *k* is relatively larger than that for *n*. This comes from the fact that most of selected materials for the training dataset in this study have the *k* value close to zero, with only a few having nonzero *k* values. As a result, the neural network is trained to fewer diverse cases for *k*, leading to relatively lower prediction accuracy compared to *n*. As expected from Table 1, higher number of optical constant cases was trained at the range between 1.35 and 1.5, resulting in higher accuracy with *n* = 1.44. The highest MSE was observed with *n* = 1.58 (1.42 × 10^−7^ for *n* and 7.06 × 10^−7^ for *k*), likely due to the low training data density in that *n* range between 1.55 and 1.60. Figure 3(b) presents the comparison of actual and predicted values for *n* = 1.334, 1.44, 1.5, and 1.58 at a 555 nm wavelength. Notably, only *n* = 1.58 case showed some difference between the actual and predicted values. Consequently, based on the overall test results and the distribution of case counts for training, the range of 1.3–1.55 can be considered the effective region for predicting optical constants. Since the *M*_convert_ result affects subsequent steps in predicting structural parameters, we evaluated the overall accuracy of structural parameter prediction after testing Steps 2 and 3. Since both Steps 2 and 3 were based on the *N*_fix_, the test was conducted with the test dataset at *N*_fix_.

**Figure 4:**
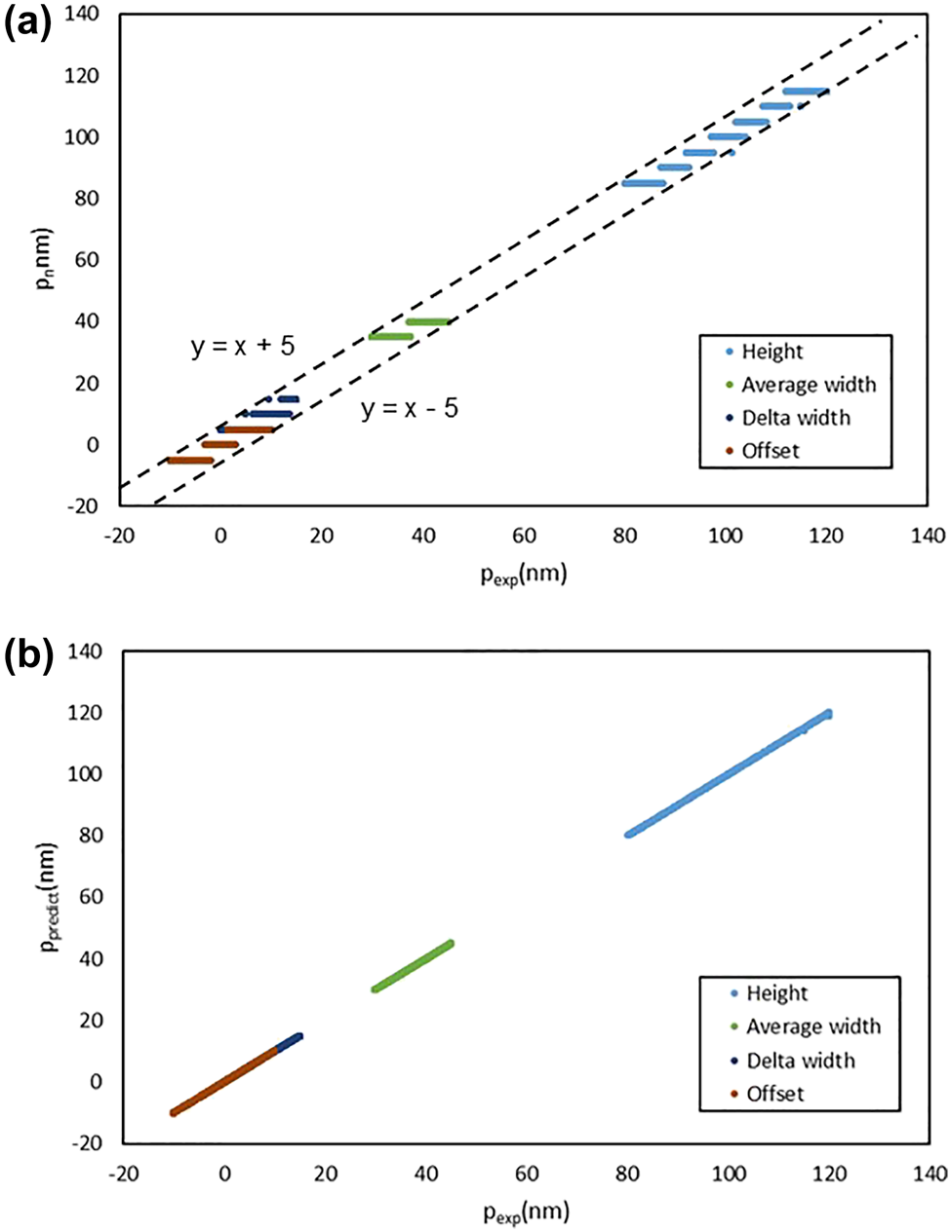
Evaluation results of structural parameter prediction performance for 660 test data: (a) in Step 2 and (b) in Step 3.

The purpose of the Step 2 neural network was to match the input MM data with the *p*_*n*_ within the same unit cell in the parameter space corresponding to *p*_exp_. If the difference between each element in the output *p* vector and the actual *p*_exp_ is not exceed 5 nm, the matching can be considered accurate. Figure 4(a) shows the distribution of *p*_*n*_ for each structural parameter, based on the Step 2 test results for 660 test data points. No output node differed by more than 5 nm, confirming accurate node matching by the neural network.

The target of Step 3 neural network is to output the relative position *p*_relative_ of *p*_exp_ compared to the matched *p*_*n*_ by using the MM data, along with the +1 and −1 node library data, as the inputs. Prediction accuracy was assessed by comparing the *p*_predict_ (= *p*_relative_ + *p*_*n*_) to the actual *p*_exp_, with smaller differences indicating more accurate prediction. Figure 4(b) illustrates the distribution of the *p*_predict_ for each structural parameter based on the test results of Step 2. In most cases, the differences were distributed within 0.1 nm, and the mean absolute error (MAE) of each structural parameter was calculated, as listed in Table 2. The overall average of MAE shows 0.028 nm. This confirmed that the structural parameter prediction algorithm in Step 2 and Step 3 performed well in terms of predicting the structural parameters of a 1D-grating specimen with a *N*_fix_.

**Table 2:** MAE for each structural parameter of 660 test data for *N*_fix_.

(nm)	Height	Average width	Delta width	Offset	Average MAE
MAE	0.024	0.025	0.037	0.026	0.028

**Figure 5:**
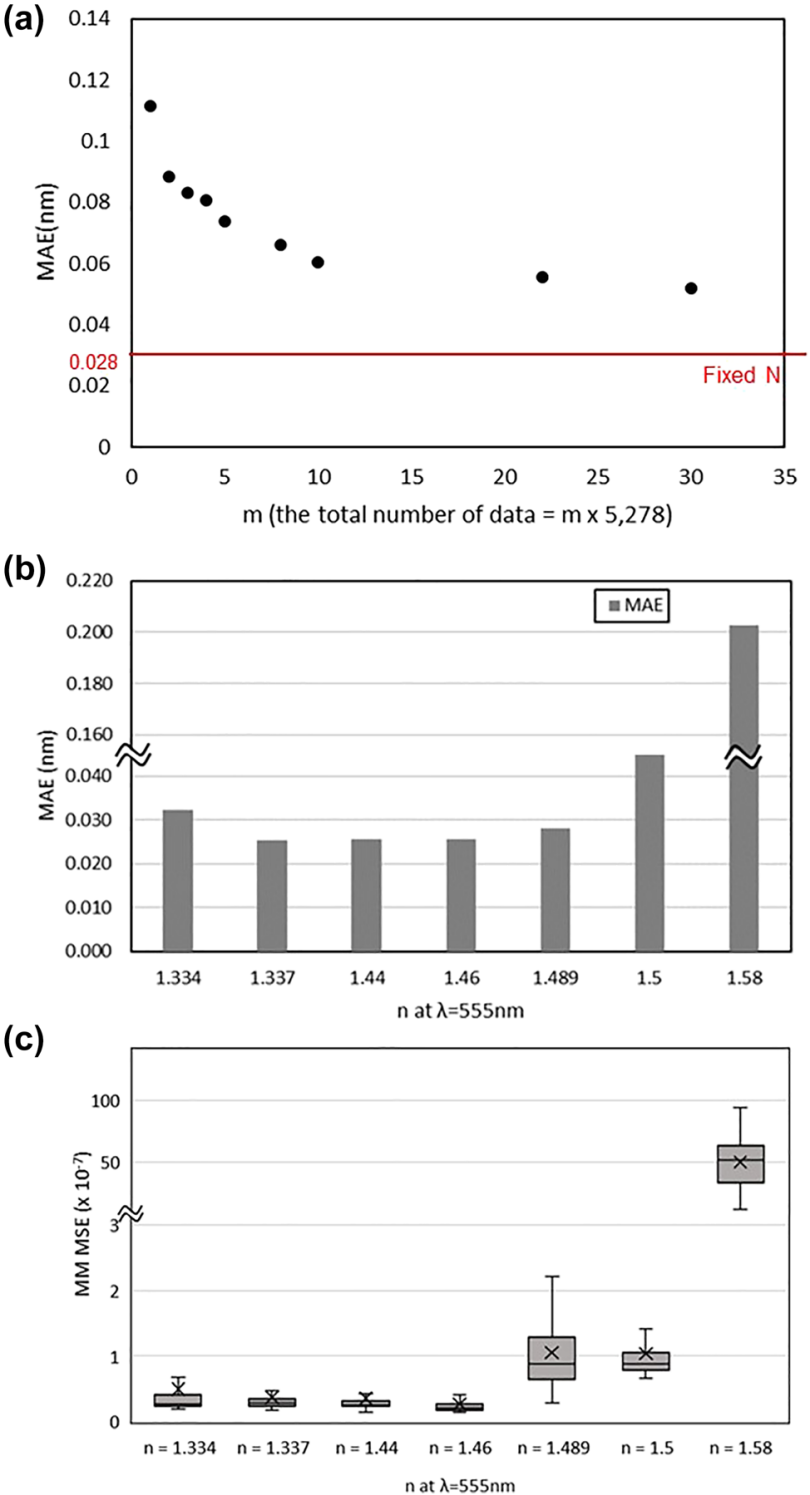
Evaluation results of prediction performance: (a) Average of MAE for each training dataset according to the number of sampling loops. (b) Average of MAE of structural parameters for 7 test optical constant cases at *m* = 30. (c) MSE of *M*_convert_ spectra of 7 test materials according to *n* at *λ* = 555 nm.

To assess the final performance of the entire three-step algorithm, we calculated the MAE of the structural parameters for each case. Step 2 and Step 3 neural networks remained constant, while the Step 1 neural network varied with the sampling loop count for each iteration to check the average of MAE on the same test dataset. Figure 5(a) shows the distribution of average MAE values of the four structural parameters based on the test results for 660 Step 1 test data points, trained with datasets generated by varying the sampling loop count (*m*). The red line labeled “fixed *N*” represents the test results obtained using the Step 2 test data with *N*_fix_, resulting in an MAE of 0.028 nm after processing through Step 2 and Step 3. The graph illustrates how the average MAE changes as training data volume increases with *m*. The average of MAE was relatively high at over 0.1 nm for *m* = 1 but decreased as *m* increased, with the rate of decrease slowing substantially after *m* = 8. At *m* = 8, the average MAE was 0.066 nm, representing a 133 % increase compared to the *N*_fix_ case but still less than 0.1 nm. At *m* = 30, the average MAE further decreased to 0.052 nm. Figure 5(b) presents the average of MAE for seven test materials when *m* = 30. Similar to Figure 3(a) for optical constant predictions, the accuracy of structural parameter predictions improves as the training data density for *n* increases.

Additionally, the accuracy of MM conversion in Step 1 was verified by comparing the MSE between the predicted *M*_convert_ spectra and the target MM spectra for *N*_fix_. As shown in Figure 5(c), the MSE was calculated for the 7 test materials. The reasonable average MSE value of 7.73 × 10^−7^ was confirmed, and when excluding the case of *n* = 1.58, the average MSE decreased to 5.96 × 10^−8^. This is because only the material of *n* = 1.58 shows *k* ≠ 0, and the rest are transparent materials with *k* = 0, suggesting that the prediction errors were relatively lower for cases with *k* = 0.

Thus, all cases except for *n* = 1.58 showed average of MAE below 0.05 nm. From the results, we confirmed that it is possible to analyze a 1D-grating specimen and accurately predict structural parameters by analyzing the measured MM data, even without repreparing the training dataset for actual optical constants of various materials. We also found that training with datasets generated from a sufficient number of sampling loops is necessary, and that prediction accuracy varies depending on the density of training optical constants in the training dataset. The analysis result for two data selected from 660 test data is described in detail in Supplementary 1. Also, the predicted optical constants and structural parameters can be used again into RCWA simulation to confirm the reliability of prediction algorithm by comparing the re-simulated MM with the measured one.

### 3.3 Experimental evaluation on the fabricated 1D SiO_2_ grating specimen

**Figure 6:**
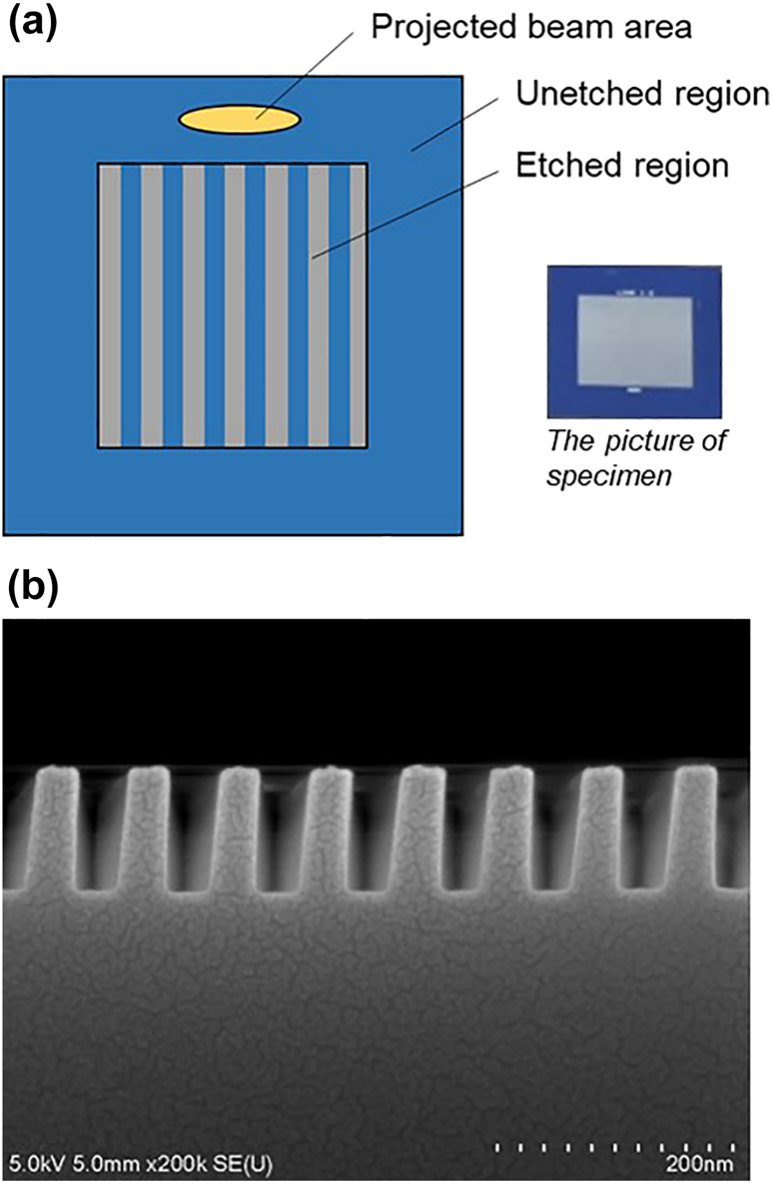
Description of the fabricated specimen: (a) Illustration of the region of fabricated specimen. (b) Cross-sectional microphotograph of scanning electron microscopy for the fabricated one-dimensional SiO_2_ grating nanostructure.

To assess the proposed algorithm by using an actual nanograting specimen, we fabricated 1D SiO_2_ grating specimen on an Si wafer through etching; its nominal values were 76 nm period, 100 nm height, 38 nm average width, 0 nm delta width, and 0 nm offset. The fabricated specimen includes both unetched region and etched region, as illustrated in Figure 6(a). The structural parameters of the etched region of this SiO_2_ nanograting were measured at 20 different points using scanning electron microscopy (SEM), and a cross-sectional imaging is shown in Figure 6(b). Additionally, we measured the optical constant of the unetched region in the SiO_2_ thin film by using a commercial SE (Woollam RC2 by J. A. Woollam Company, Nebraska). These extracted optical constants and the structural parameters corresponding to the 20 different points were used as the simulation inputs to generate 20 MM data. The generated MM data were subsequently used to assess the analysis algorithm developed in this study. To compare the accuracy of the proposed algorithm, we also conducted an analysis using a conventional approach under the same conditions. The conventional approach employed an iterative Levenberg–Marquardt (LM) regression algorithm, comparing the MM calculated in each iteration of the RCWA simulation to the measured MM until the MSE was minimized [35], [36], [37]. A detailed explanation of the conventional approach is provided in Supplementary 2 [38], [39].

**Figure 7:**
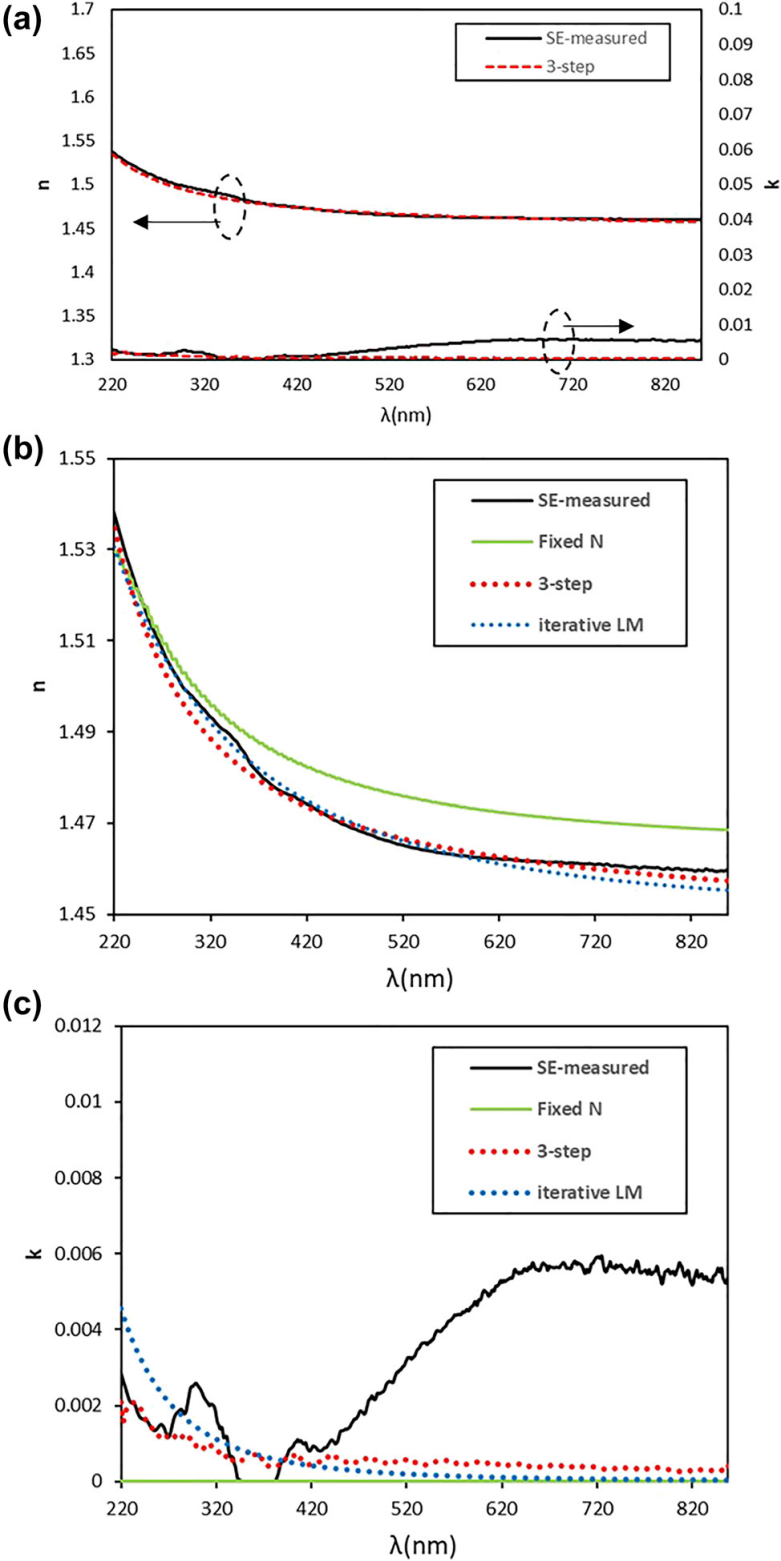
Predicted results for optical constants: (a) Comparison of the measured optical constants of SiO_2_ thin film and the predicted optical constants by the three-step algorithm, and comparison of (b) the measured refractive index and (c) extinguish coefficient with the predicted values by different analysis algorithms.

The analysis was performed using the conventional approach (iterative LM algorithm) and the proposed algorithm (three-step algorithm) in identical computational environments and by using the same resources as those employed for training. Table 3 presents a comparison of the analysis algorithm results obtained using iterative LM algorithm and the proposed three-step algorithm. The table includes the mean values and 95 % confidence intervals for each structural parameter based on SEM measurements, as well as the mean values of each structural parameter analyzed at the 20 points by using each algorithm. The results of the three-step algorithm were within the confidence interval for all parameters, whereas the results of the iterative LM algorithm were within the confidence interval for all parameters, except for delta width. To confirm the role of Step 1 to predict structural parameters in the proposed algorithm, the MAE values were also evaluated through a special process without Step 1 from the prediction process. As compared in Table 3, the MAE from the 3-step algorithm resulted in lower values across all structural parameters, compared to those from the special process without Step 1, demonstrating that each step in the proposed algorithm has a role to predict the structural parameters accurately. Furthermore, the average analysis time of the three-step algorithm of 132 ms was considerably shorter than that of iterative LM algorithm of 7.3 h. Next, we compared the optical constants obtained using both algorithms. Figure 7(a) presents a comparison of the *n* and *k* data obtained from SE measurements and the optical constants output by Step 1 of the three-step algorithm. The refractive index (*n*) exhibited a high degree of agreement, while the extinction coefficient (*k*) values exhibited relatively good agreement below 420 nm, although some difference was shown across the entire range. Figure 7(b) and (c) presents a comparison of the *n* and *k* values obtained from the SE measurements, the *N*_fix_ assumption in the three-step algorithm, and the outputs of both algorithms. When comparing the *n* and *k* values across the entire wavelength range using MSE, the three-step algorithm yielded *n* and *k* values of 4.69 × 10^−6^ and 1.29 × 10^−5^, respectively, while iterative LM algorithm yielded *n* and *k* values of 5.26 × 10^−6^ and 1.47 × 10^−5^. Both algorithms exhibited reasonable accuracy for predicting optical constants, with the MSE obtained using the three-step algorithm being slightly lower.

**Table 3:** Comparison on the predicted values for the 1D SiO_2_ grating with those observed by SEM.

(nm)	Height	Average width	Delta width	Offset
Nominal value	100.000	38.000	0.000	0.000
SEM	104.95 ± 0.64	39.7 ± 0.71	5.5 ± 0.77	7.39 ± 0.46
Mean value (iterative LM)	105.39	39.47	6.35	7.66
Mean value (3-step)	105.01	39.62	5.31	7.67
MAE (3-step)	0.065	0.075	0.187	0.083
*MAE (without step 1)* ^a^	*0.093*	*0.298*	*0.537*	*0.317*

^a^This means MAE value prepared from the special process without Step 1 for comparison.

**Figure 8:**
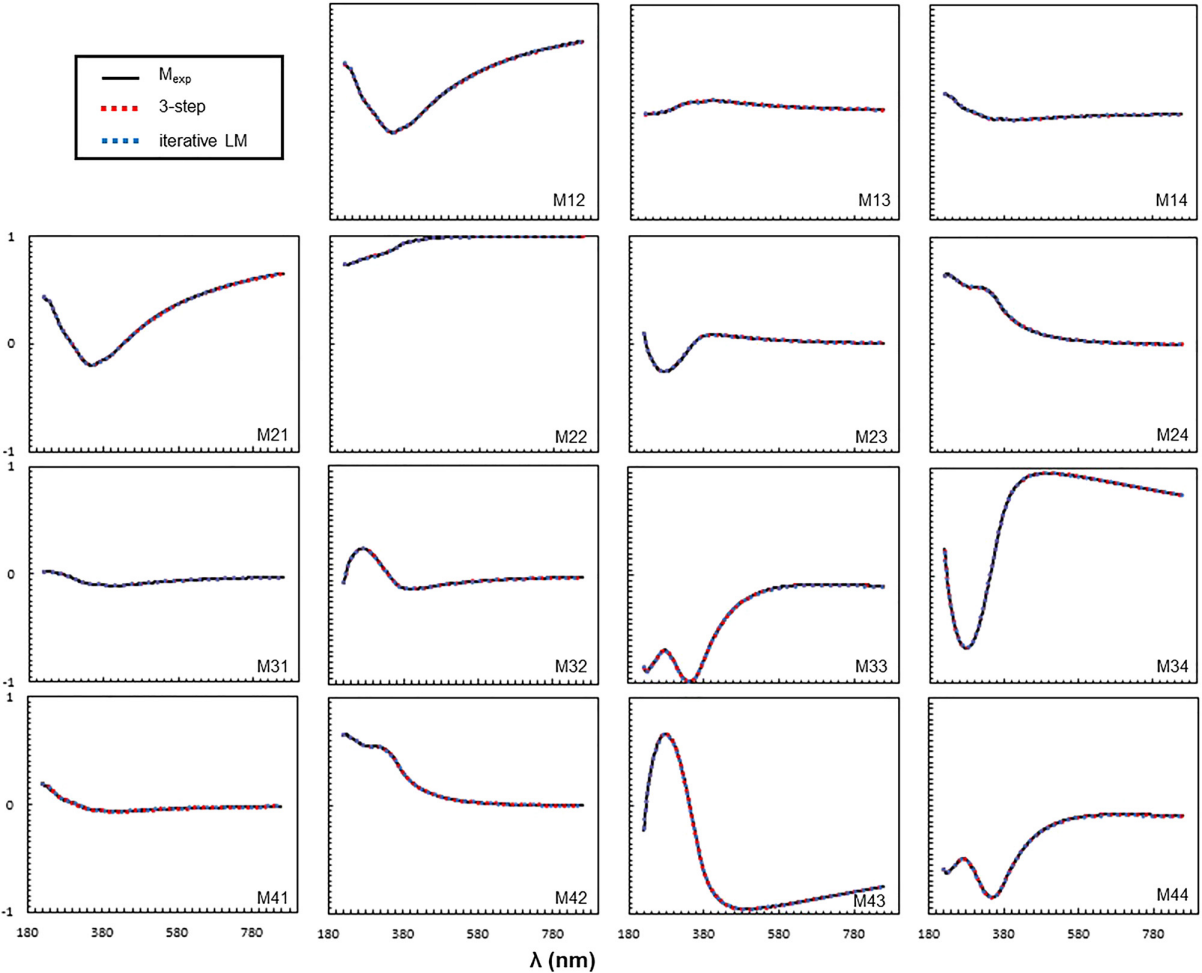
Comparison of the calculated MM spectra from three-step algorithm and iterative LM algorithm with the measured MM spectra at an azimuthal angle of 45°.

Finally, we compared the calculated MMs obtained using both algorithms to the measured input MM in terms of MSE. For the iterative LM algorithm, the MM obtained from the final fitting result was compared to the measured MM, while for the three-step algorithm, the simulated MM obtained using the predicted *N* and *p* as the inputs was compared to the measured MM, and the MSE obtained through this process can serve as an indicator of the reliability of the predicted *p* from the three-step algorithm. The average of MSE obtained from each algorithm as 9.07 × 10^−6^ for the three-step algorithm and 1.19 × 10^−5^ for the iterative LM algorithm, with both algorithms showing low levels of MSE. Figure 8 presents a comparison of the MM spectra of one specific point for both algorithms, demonstrating strong agreement across the entire wavelength range with the measured data.

In conclusion, for the fabricated 1D SiO_2_ grating specimen, the three-step algorithm demonstrated analysis accuracy comparable to the conventional iterative LM algorithm, while its computation time was considerably shorter. In the case of iterative LM algorithm, the selection of an appropriate optical model and initial values has a significant impact on the accuracy of the analysis. In contrast, the proposed algorithm does not rely on assumptions about the optical model and does not require initial values. Since there are lots of experimental variations that affect the MM data evaluation for actual fabricated specimens, it is still required to improve our proposed analysis algorithm by incorporating more environmental variables and measurement noises. Additionally, since the neural network model is trained on wavelength-dependent optical constants rather than data based on a few values applied to a specific optical model, it is expected to offer greater reliability in analysis. The proposed three-step analysis algorithm, which consists of three individual neural networks, can accurately predict the structural parameters of nanostructures even when the optical constants are unknown due to different materials or any changes in the fabrication and measurement conditions. In addition, this algorithm has another advantage for the applications where fast analysis is required.

## 4 Conclusions

We proposed a three-step algorithm for rapid and accurate extraction of the structural parameters from the spectroscopic ellipsometry (SE) data, achieving high-speed analysis time by replacing the least-square iterative computations with neural networks, without repreparing the training dataset for actual optical constants of the material. The proposed algorithm obtains optical constants first and accurately predicts structural parameters by progressively narrowing the prediction range at each step based on separate neural networks trained on simulation data. The newly developed algorithm was validated using both simulation data and the SEM data of fabricated 1D SiO_2_ nanogratings with 38 nm width; the results confirm both high accuracy with average mean absolute error (MAE) of 0.103 nm and fast analysis speed of 132 ms in extracting the structural parameters of nanogratings. Also the proposed algorithm is expected to facilitate a fast nondestructive analysis through MM-based SE and shows flexibility in handling various optical constants due to different materials or any changes in the fabrication and measurement conditions. When needed, the reliability of the predicted structural parameters can be validated further in the algorithm, thereby checking the reliability of the results. Thus, the proposed algorithm can serve as a more efficient solution in the industrial and research fields related to the SE measurement, where both fast and accurate analysis on nanostructures are required, particularly in applications demanding real-time monitoring or high-throughput analysis.

## Supplementary Material

Supplementary Material Details
