# Supplementary material for: Neural network-based analysis algorithm on Mueller matrix data of spectroscopic ellipsometry for the structure evaluation of nanogratings with various optical constants
Source: Nanophotonics. 2025 Feb 12;14(4):471–84. doi: 10.1515/nanoph-2024-0565 (PMC11834052; doi:10.1515/nanoph-2024-0565)
Supplement: Supplementary file 1 — Supplementary Material Details [file j_nanoph-2024-0565_suppl_001.docx]

Juwon Jung, Nagyeong Kim, Kibaek Kim, Jongkyoon Park, Yong Jai Cho, Won Chegal^*^, and Young-Joo Kim^[[1]](#footnote-1)^

Neural network-based analysis algorithm on mueller matrix data of spectroscopic ellipsometry for the structure evaluation of nano-gratings with various optical constants: Supplement

1. **Test results for the simulation data of two optical constants**

For two cases where n significantly different from Nfix (case1: lower than Nfix, case2: higher than Nfix), the results of the three step algorithm (optical constant prediction, converted MM prediction, and structural parameter prediction) are presented as follows. From these results, it can be observed that successful structural parameter prediction is possible even for materials with optical constants that greatly differ from Nfix.

**Case 1) Lower than Nfix**

**material: n=1.334 at 555nm with 1D grating structure of 76nm period**
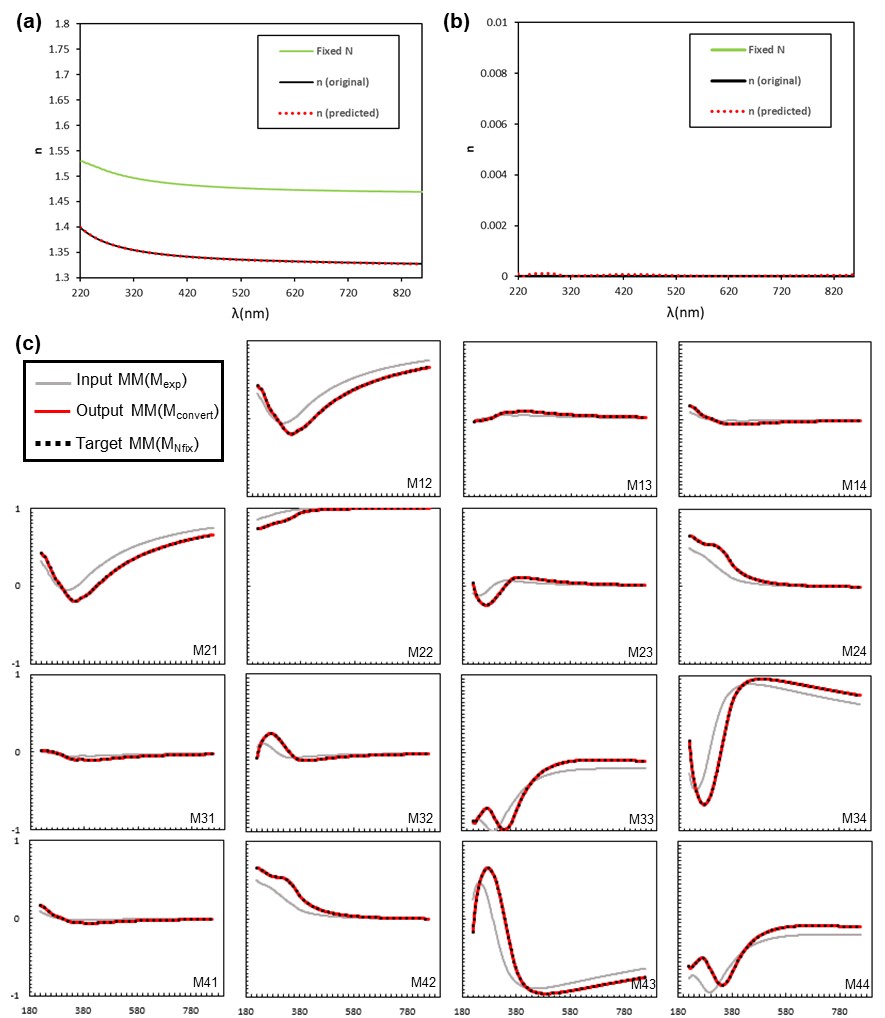


**Fig. S1:** Step 1's prediction result for case 1 (a) prediction for n (b) prediction for k (c) prediction for converted MM.

**Tab. S1:** Structural parameter prediction results for case 1.

| **(nm)** | **Height** | **Average width** | **Delta width** | **Offset** |
| --- | --- | --- | --- | --- |
| **real value** | 105.383 | 38.759 | 1.287 | -3.339 |
| **Predicted value** | 105.358 | 38.759 | 1.303 | -3.332 |
| **MAE** | 0.025 | 0 | 0.016 | 0.007 |

**Case 2) Higher than Nfix**

**material: n=1.58 at 555nm with 1D grating structure of 76nm period**
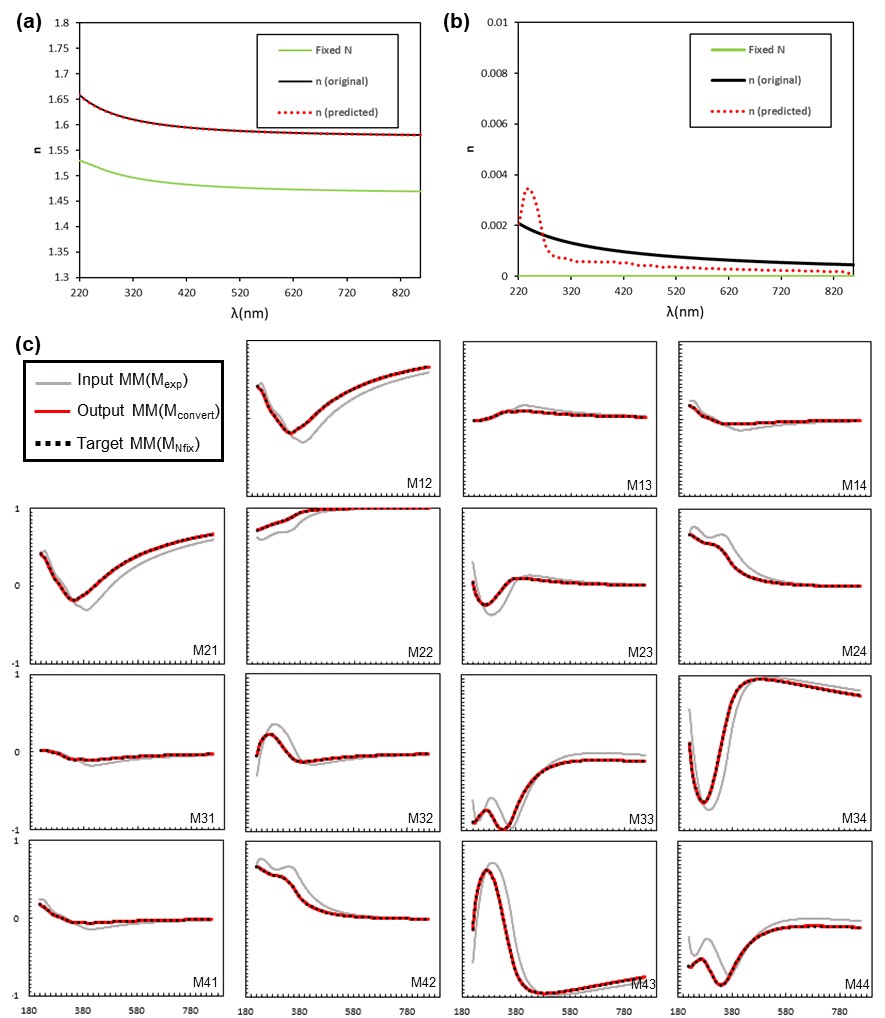


**Fig. S2:** Step 1's prediction result for case 2 (a) prediction for n (b) prediction for k (c) prediction for converted MM.

**Tab. S2:** Structural parameter prediction results for case 2.

| **(nm)** | **Height** | **Average width** | **Delta width** | **Offset** |
| --- | --- | --- | --- | --- |
| **real value** | 105.817 | 37.584 | 2.209 | 1.499 |
| **Predicted value** | 105.791 | 37.846 | 2.639 | 1.543 |
| **MAE** | 0.026 | 0.262 | 0.43 | 0.044 |

1. **Optical Model for iterative LM analysis algorithm**

The conventional algorithm employed an iterative Levenberg-Marquardt (LM) regression algorithm, comparing the MM calculated in each iteration of the RCWA simulation to the measured MM. The fitting parameters included four structural parameters—height, average width, delta width, and offset—and six parameters from the optical model of the optical constants, which was based on the Cauchy model, resulting in a total of 10 fitting parameters [37, 38]. The defined Cauchy model is expressed in Eq. S1, and the initial values must be manually specified. We set the initial fitting parameters as follows: (height, average width, delta width, offset, p_1_, p_2_, p_3_, p_4_, p_5_, p_6_) = (100 nm, 38 nm, 0 nm, 0 nm, 1.447, 0.366, 0, 0, 0, 0).

| $n\left( \lambda\right)=p_{1}+\frac{{10}^{4}\cdot p_{2}}{\lambda^{2}}+\frac{{10}^{9}\cdot p_{3}}{\lambda^{4}}, k\left( \lambda\right)={{10}^{-5}\cdot p}_{4}+\frac{{10}^{4}\cdot p_{5}}{\lambda^{2}}+\frac{{{10}^{9}\cdot p}_{6}}{\lambda^{4}},$ | (S1) |
| --- | --- |

The loss function for the optimization process is the mean square error (MSE), and the parameter values that minimize this loss function are ultimately output.

1. Juwon Jung and Nagyeong Kim contributed equally to this work as shared first authors.

   **Corresponding authors:** **Young-Joo Kim**, Department of Mechanical Engineering, Yonsei University, Seoul, Republic of Korea; yjkim40@yonsei.ac.kr; <https://orcid.org/0000-0002-5378-422X>; and **Won Chegal**, Semiconductor and Display Metrology Group, Korea Research Institute of Standards and Science, 267 Gajeongno, Yuseong-Gu, Daejeon, 34113, Republic of Korea and Graduate School of Analytical Science and Technology (GRAST), Chungnam National University, Daejeon, 34134, Republic of Korea; wchegal@kriss.re.kr; https://orcid.org/0000-0001-8774-8318

   **Juwon Jung,** **Nagyeong Kim and Kibaek Kim**, Department of Mechanical Engineering, Yonsei University, Seoul, Republic of Korea

   **Jongkyoon Park, Yong Jai Cho**, Semiconductor and Display Metrology Group, Strategic Technology Research Institute, Korea Research Institute of Standards and Science, Daejeon, Republic of Korea [↑](#footnote-ref-1)
